# Novel nose poke-based temporal discrimination tasks with concurrent in vivo calcium imaging in freely moving mice

**DOI:** 10.1186/s13041-019-0515-7

**Published:** 2019-11-06

**Authors:** William D. Marks, Hisayuki Osanai, Jun Yamamoto, Sachie K. Ogawa, Takashi Kitamura

**Affiliations:** 1 Department of Psychiatry, University of Texas Southwestern Medical Center, Dallas, TX 75390 USA; 2 Department of Neuroscience, University of Texas Southwestern Medical Center, Dallas, TX 75390 USA

**Keywords:** Time, Timing, Temporal discrimination, Space, Memory, Hippocampus, Time cell, Entorhinal cortex, In vivo calcium imaging

## Abstract

The hippocampus has been known to process temporal information as part of memory formation. While time cells have been observed in the hippocampus and medial entorhinal cortex, a number of the behavioral tasks used present potential confounds that may cause some contamination of time cell observations due to animal movement. Here, we report the development of a novel nose poke-based temporal discrimination task designed to be used with in vivo calcium imaging for the analysis of hippocampal time cells in freely moving mice. First, we developed a ten second held nose poke paradigm for use in mice to deliver a purer time metric for the analysis of time cell activity in hippocampus CA1. Second, we developed a temporal discrimination task that involves the association of held nose poke durations of differing lengths with differential spatial cues presented in arms on a linear I-maze. Four of five mice achieved successful temporal discrimination within three weeks. Calcium imaging has been successfully performed in each of these tasks, with time cell activity being detected in the 10s nose poke task, and calcium waves being observed in discrete components of the temporal discrimination task. The newly developed behavior tasks in mice serve as novel tools to accelerate the study of time cell activity and examine the integration of time and space in episodic memory formation.

## Main text

While the hippocampus (HPC) is traditionally known for processing spatial information [1], it also processes temporal information which is important for the formation of episodic memories [2–5]. Neurons that repeatedly fire at a specific moment during the delay period of behavior tasks have been found in the hippocampus and medial entorhinal cortex (EC), and are referred to as time cells [3–7]. Time cell activity has been observed during the delay periods of treadmill running tasks, trace eye blink conditioning, spatial working memory tasks, timing tasks in virtual reality and of delayed matching to sample tasks [3, 6–11]. The activity of these time cells and their sequences during delay or interval periods is considered to link events in time, and remember specific timing in memory formation [3–5]. While time cells can be identified during the delay periods of timing-related behavior tasks even with animal body movement, it would be more desirable to analyze time cell activity in an immobile condition [8–10, 12].

**Fig. 1:**
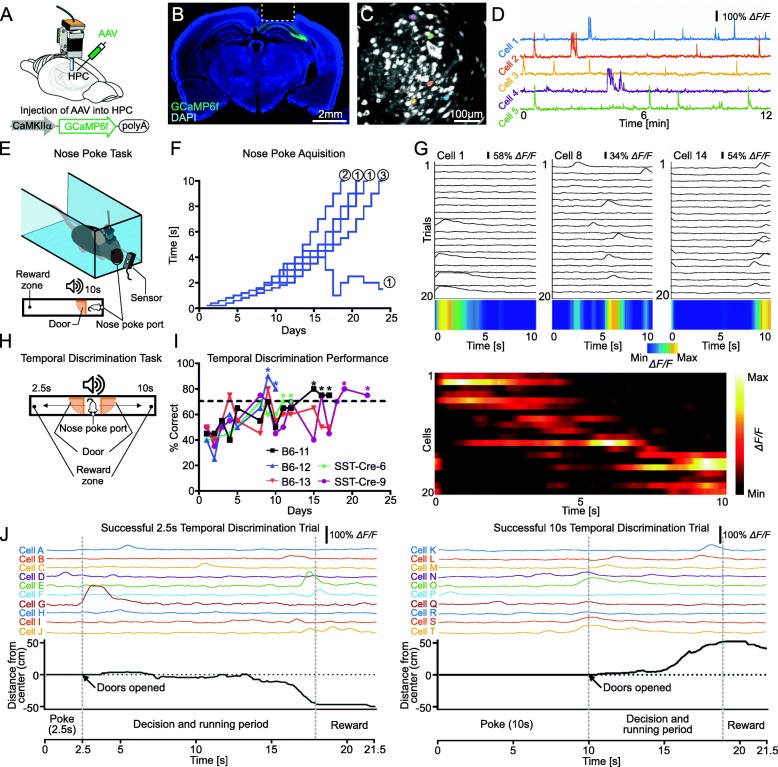
Temporal discrimination task with in vivo calcium imaging in freely moving mice. **a)** Viral injection and lens implantation into dorsal hippocampus. **b)** Post-surgical histology of implanted mice. Expression of GCaMP6f was observed in dorsal hippocampal CA1 and CA2 (green). DAPI: blue. Lens placement was just above the hippocampus. The implant consisted of a GRIN lens (1 mm diameter) contained within a glass bottomed cannula. **c)** Stacked GCaMP6f image acquired through the miniature fluorescent microscope over 10 min of imaging from CA1 at 20 Hz. Specifically noted cells correspond to traces in **d**. **d)** Relative fluorescence changes (*ΔF/F* %) for five hippocampal CA1 cells identified in **c**. **e)** Nose poke behavior apparatus. Mice are trained to hold a 10 s nose poke at a nose poke port in a gated chamber (width; 8 cm, length 14 cm, height 3 cm) at the start of a linear track (Width; 8 cm, length; 30 cm, height, 3 cm). Nose poke activity was detected by an infrared photointerrupter module positioned adjacent to the nose poke port exterior opening. After holding the nose poke for the required amount of time, the door opens (orange), allowing access to the reward zone at the end of the track. **f)** Long-duration nose poke learning curve. Each blue line indicates the performance of individual mice. Numbers at the end of each line indicate the number of mice represented by that terminus as several of the mice had overlapping performance levels at various points in the training procedure. **g) Upper panel;** Three examples of hippocampal time cells imaged during successful nose poke intervals. Traces show the relative fluorescence changes (*ΔF/F* %) across multiple 10s held poke trials for each cell. The color code plot at the bottom represents the averaged trace for each cell. **Lower panel;** Simultaneously imaged neurons from a single mouse (SST-Cre-9). Each row represents the normalized firing rate (5 ms bins) for one single neuron over the nose poke period averaged over all trials for each cell that met the criterion for time cells. Identified time cells were sorted by their peak firing time. **h)** Nose poke-based temporal discrimination behavior apparatus. Mice were trained to associate a 2.5 s held nose poke with a left or right turn (randomized per mouse), and a 10s poke with the opposite direction. The apparatus consists of a central nose poke port-equipped starting box (width; 8 cm, length 10 cm, height 3 cm) with two doors (orange) and two arms (width; 8 cm, length 50 cm, height 3 cm) on the left and the right. **i)** Learning curves for the nose poke-based temporal discrimination task in mice. Each line indicates the performance of 1 mouse; Three C57BL6J mice (B6–11, B6–12, B6–13), and Two SST-Cre transgenic mice (SST-Cre-6, SST-Cre-9). Dashed line indicates 70% threshold of statistical significance (Binomial test, *P* < 0.05). * indicates 70% or higher success rate on 2 consecutive days. **j)** Calcium imaging during the temporal discrimination task. Relative fluorescence change (*ΔF/F* %) for a total of 20 (out of 98 imaged) cells have been presented for successful trials associated with a 2.5 s nose poke (left panel, cells A-J) and a 10s nose poke (right panel, cells K-T). The trace activity data has been temporally aligned with movement tracking data showing the distance in cm of the animal’s head from the center point (nose poke port) of the I-maze. Vertical dashed lines delineate discrete periods of the trials

In this study, we aim to deliver a purer temporal metric for the analysis of time cells with minimal contamination from body movement, speed, or head direction, which will be integrated with time by the HPC-EC circuit, while also depending on an attention-requiring task, rather than a passive wait period. To achieve this, we have designed a task in which mice are trained to actively hold a nose poke for 10 s in order to open a door leading to a reward zone. This task is designed to be paired with in vivo calcium imaging from hippocampal CA1 pyramidal cells during the held nose poke period in order to identify time cell activity in larger ensembles of neurons simultaneously. All animal lines were obtained from Jackson labs and bred in house. Five 26–27 week-old C57BL6J male mice (Jax 000664) and three 22–29 week-old Somasostatin (SST)-Cre transgenic male mice on a C57BL6J background (Jax 028864) were injected with 500 nl of adeno-associated virus 5 (AAV5)-calcium/calmodulin-dependent protein kinase II alpha (CaMKIIα):GCaMP6f, a calcium indicator (obtained from UPENN Vector Core), in the right dorsal hippocampal CA1 region (AP:-2.15, ML:+ 1.5, DV:-1.3). Two weeks after the injection, mice were implanted with a micro gradient-index (GRIN) lens (1 mm diameter, 4 mm length, Inscopix) targeting the right dorsal hippocampal CA1 region (Fig. 1a-b). GCaMP6f signals of individual hippocampal CA1 cells were imaged by using a miniaturized, head-mounted fluorescence microscope (Inscopix) (Fig. 1c-d) [13–15]. Following a recovery period after surgery including baseplate attachment to the head, mice were placed on food restriction and maintained at 80% of their starting weight. Following one week of food restriction and handling, mice were habituated to the behavioral apparatus for two days prior to training (Fig. 1e, Additional file 1: Figure S1). All behavior experiments were performed during the light cycle. Video recordings made using a Noldus camera. Mice were initially required to hold a 200 ms poke to open the door, which can be achieved by simple investigation of the nose poke chamber. If mice removed their nose from the poke port at any time before the required duration is reached, the timer was restarted. Over two to three days the mice learned to associate the nose poke and paired 1500 Hz tone with the opening of the door and subsequent reward consisting of a small pellet of their standard chow weighing 0.05–0.1 g. On day 3–4, mice were expected to make 5 trials at 200 ms, then the duration was increased to 400 ms for 5 trials. The duration was increased in 200 ms intervals daily until a 1 s poke was achieved. At that point, daily increases were at a 500 ms interval until a 5 s poke was reached, and from that point a 1 s interval was applied until the 10s poke was achieved. If an animal failed to successfully extend the nose poke duration on any given day, that day’s protocol was repeated the next day without further increase until the original threshold was met. Once the 10s threshold was reached, mice reliably performed the 10s nose poke for at least 20 trials/hr. Importantly, minimal movement of the body, tail, and limbs was observed in the trained mice during pokes. Head motion was also limited except occasional chewing on the edge of the nose poke hole and slight rolling of the head. Any mouse that failed to acquire the 200 ms nose poke in 4 days or to achieve the 10s poke by 28 days was removed from the study. Our success rate for the nose poke task in lens-implanted mice was 88% (7 of 8 animals) (Fig. 1f). Training for the 7 successful mice took between 18 and 24 days to reach the 10s threshold (Fig. 1f).

After nose poke training, we then examined the somatic calcium activity (% *ΔF/F*) of individual hippocampal CA1 pyramidal cells during the nose poke task. Calcium activity was captured at 20 Hz on a miniature fluorescent microscope. Single unit calcium activity from hippocampal CA1 pyramidal cells was isolated by using Inscopix Data Processing Software v1.2 and ImageJ, as previously examined [13–15] (Fig. 1d,g). We obtained individual calcium activity from 302 cells across 3 mice (90, 50, and 162 = 302 cells in total) during the nose poke task, indicating that we successfully developed the nose poke task with in vivo calcium imaging in freely moving mice. Then, we analyzed individual calcium activity during successful nose poke periods to identify time cell activity. We observed 22% of hippocampal CA1 pyramidal cells that were repeatedly activated at a specific moment during successful nose poke periods in at least one fifth of the total trials (total of 90 cells were imaged from mouse "SST-Cre-9", Fig. 1g.). Some neurons had phasic responses within the first 2 s (6/20), and others activated later with responses at the middle (seconds 2–5, 5/20; seconds 5–8, 2/20) or end (seconds 8–10, 7/20) of that period (Fig. 1g, upper). We also characterized time cells as an ensemble of neurons imaged simultaneously in a single mouse, and observed that the mean peak of calcium activity for each time cell occurred at sequential moments and that the ensemble of time cells bridges the entire nose poke period (Fig. 1g, lower).

We then further developed a nose poke-based temporal discrimination task in mice. Mice which were previously trained for the 10s nose poke task were used in this task. Five mice were habituated to the new apparatus for two days. The apparatus consists of a nose poke port located between two linear arms which are gated with mechanical doors. Each of the arms had unique spatial features on the floor, with the left arm having vertical black and white stripes 3 cm thick, and the right having black polka dots 1.5 cm in diameter on a white background. The walls of the room had additional unique visual cues comprised of shapes and patterns made from vertical or horizontal strips of tape (Fig. 1h, Additional file 1: Figure S1). Mice were trained to associate a 2.5 s tone with one arm, and a 10s tone with the other for 7 days. After training, we started the temporal discrimination task in which mice were randomly given a short or long poke threshold (Fig. 1i). In the temporal discrimination task, upon reaching the threshold of the randomly assigned 2.5 or 10 s nose poke, both doors open and the mouse must choose which arm is associated with the poke duration it performed to open the doors. Each mouse was given 20 trials per day, with four training repetitions given prior to each testing period. The statistical threshold for considering successful acquisition of temporal discrimination was set at a 70% correct response rate for 20 trials (Binomial test, *P* < 0.05) on two consecutive days. Four of five mice were observed to successfully discriminate within 22 days (Fig. 1i). Furthermore, we successfully obtained individual calcium activity during the nose poke, running and reward periods in the temporal discrimination task (Fig. 1j).

In this study, we developed novel nose poke-based temporal discrimination task with in vivo calcium imaging in freely moving mice. While time cells have been observed using head-fixed animals [8, 9], we believe that our task in freely moving mice can be useful to study the neural mechanisms of temporal discrimination, and furthermore will provide added variety to the extant behavioral approaches for examining the integration of time and space with the application of transgenic strains and viral manipulations.

## Supplementary information

**Additional file 1: Figure S1A.** Basic nose poke apparatus consisting of a gated start chamber with a nose poke port within a linear track (left panel). The Arduino unit serving as the processing unit and main output of nose poke timing and experimental logs is shown in the upper right. The center panels show close up views of the nose poke port, and the photointerrupter module used as the nose poke sensor. The surface above the nose poke port prevents the mouse from climbing over and interacting with the photointerrupter from the opposite side. The door and servo mechanism are shown in the right panel. The door has extended surfaces past the sides of the maze to prevent the mouse from climbing around it. **B)** Layout of the I-maze used in temporal discrimination testing. Each of the arms has a unique spatial cue associated with it. The nose poke port (inset) and door mechanisms are similar to those in the basic nose poke task. Highlighted here are other common features with the nose poke task. A speaker is used to deliver simultaneous auditory cues during active pokes. A TTL output delivers time stamp data to the nVoke system temporal allowing linkage between nose pokes and calcium spike events. A rheostat is used for program selection, and programs are triggered and terminated using the manual triggers.

## Data Availability

Data and scripts for analysis and behavioral apparatus operation will be made available by the corresponding author upon reasonable request.

## Abbreviations

AAV: Adeno-associated virus
CaMKIIα: Calcium/calmodulin-dependent protein kinase II alpha
EC: Entorhinal cortex
GRIN lens: Gradient-Index lens
HPC: Hippocampus
SST: Somatostatin

## References

[1] Eichenbaum H, Dudchenko P, Wood E, Shapiro M, Tanila H (1999). The hippocampus, memory, and place cells: is it spatial memory or a memory space?. Neuron..

[2] Kitamura T, Pignatelli M, Suh J, Kohara K, Yoshiki A, Abe K (2014). Island cells control temporal association memory. Science..

[3] Eichenbaum H (2014). Time cells in the hippocampus: a new dimension for mapping memories. Nat Rev Neurosci.

[4] Kitamura T, Macdonald CJ, Tonegawa S (2015). Entorhinal-hippocampal neuronal circuits bridge temporally discontiguous events. Learn Mem.

[5] Kitamura T (2017). Driving and regulating temporal association learning coordinated by entorhinal-hippocampal network. Neurosci Res.

[6] Pastalkova E, Itskov V, Amarasingham A, Buzsaki G (2008). Internally generated cell assembly sequences in the rat hippocampus. Science..

[7] MacDonald CJ, Lepage KQ, Eden UT, Eichenbaum H (2011). Hippocampal "time cells" bridge the gap in memory for discontiguous events. Neuron..

[8] Modi MN, Dhawale AK, Bhalla US (2014). CA1 cell activity sequences emerge after reorganization of network correlation structure during associative learning. Elife..

[9] MacDonald CJ, Carrow S, Place R, Eichenbaum H (2013). Distinct hippocampal time cell sequences represent odor memories in immobilized rats. J Neurosci.

[10] Heys JG, Dombeck DA (2018). Evidence for a subcircuit in medial entorhinal cortex representing elapsed time during immobility. Nat Neurosci.

[11] Sabariego M, Schonwald A, Boublil BL, Zimmerman DT, Ahmadi S, Gonzalez N (2019). Time cells in the Hippocampus are neither dependent on medial Entorhinal cortex inputs nor necessary for spatial working memory. Neuron..

[12] Halberstadt AL, Sindhunata IS, Scheffers K, Flynn AD, Sharp RF, Geyer MA (2016). Effect of 5-HT2A and 5-HT2C receptors on temporal discrimination by mice. Neuropharmacology..

[13] Kitamura T, Sun C, Martin J, Kitch LJ, Schnitzer MJ, Tonegawa S (2015). Entorhinal Cortical Ocean cells encode specific contexts and drive context-specific fear memory. Neuron..

[14] Sun C, Kitamura T, Yamamoto J, Martin J, Pignatelli M, Kitch LJ (2015). Distinct speed dependence of entorhinal island and ocean cells, including respective grid cells. Proc Natl Acad Sci U S A.

[15] Kitamura T, Ogawa SK, Roy DS, Okuyama T, Morrissey MD, Smith LM (2017). Engrams and circuits crucial for systems consolidation of a memory. Science..

