# Supplementary material for: Novel nose poke-based temporal discrimination tasks with concurrent in vivo calcium imaging in freely moving mice
Source: Mol Brain. 2019 Nov 6;12:90. doi: 10.1186/s13041-019-0515-7 (PMC6836444; doi:10.1186/s13041-019-0515-7)
Supplement: Supplementary file 1 — Additional file 1: Figure S1A. Basic nose poke apparatus consisting of a gated start chamber with a nose poke port within a linear track (left panel). The Arduino unit serving as the processing unit and main output of nose poke timing and experimental logs is shown in the upper right. The center panels show close up views of the nose poke port, and the photointerrupter module used as the nose poke sensor. The surface above the nose poke port prevents the mouse from climbing over and interacting with the photointerrupter from the opposite side. The door and servo mechanism are shown in the right panel. The door has extended surfaces past the sides of the maze to prevent the mouse from climbing around it. B) Layout of the I-maze used in temporal discrimination testing. Each of the arms has a unique spatial cue associated with it. The nose poke port (inset) and door mechanisms are similar to those in the basic nose poke task. Highlighted here are other common features with the nose poke task. A speaker is used to deliver simultaneous auditory cues during active pokes. A TTL output delivers time stamp data to the nVoke system temporal allowing linkage between nose pokes and calcium spike events. A rheostat is used for program selection, and programs are triggered and terminated using the manual triggers. [file 13041_2019_515_MOESM1_ESM.docx]

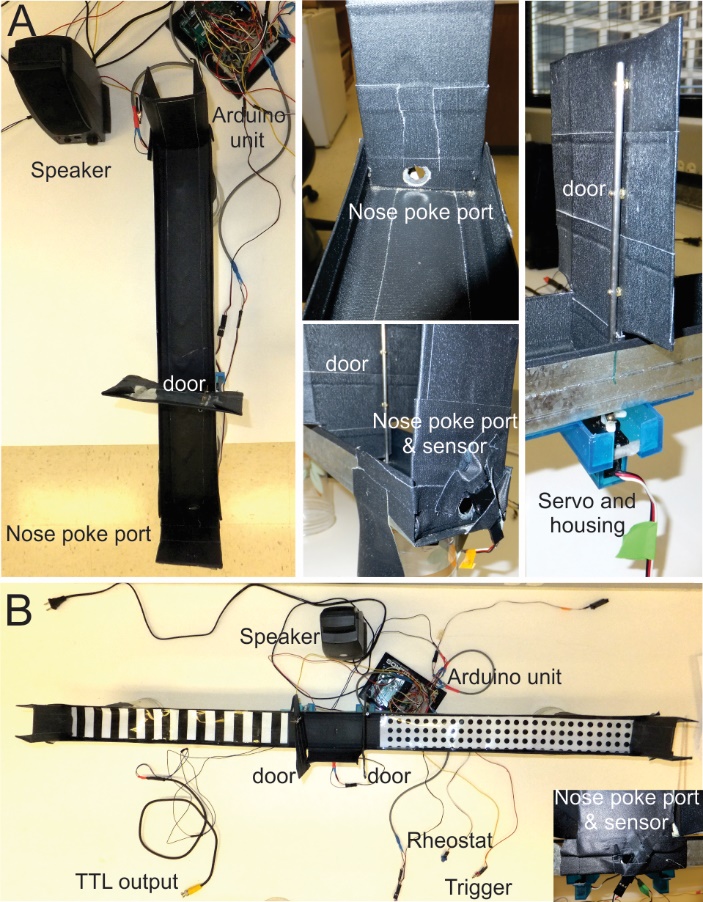


**Supplemental Figure 1**. **A)** Basic nose poke apparatus consisting of a gated start chamber with a nose poke port within a linear track (left panel). The Arduino unit serving as the processing unit and main output of nose poke timing and experimental logs is shown in the upper right. The center panels show close up views of the nose poke port, and the photointerrupter module used as the nose poke sensor. The surface above the nose poke port prevents the mouse from climbing over and interacting with the photointerrupter from the opposite side. The door and servo mechanism are shown in the right panel. The door has extended surfaces past the sides of the maze to prevent the mouse from climbing around it. **B)** Layout of the I-maze used in temporal discrimination testing. Each of the arms has a unique spatial cue associated with it. The nose poke port (inset) and door mechanisms are similar to those in the basic nose poke task. Highlighted here are other common features with the nose poke task. A speaker is used to deliver simultaneous auditory cues during active pokes. A TTL output delivers time stamp data to the nVoke system temporal allowing linkage between nose pokes and calcium spike events. A rheostat is used for program selection, and programs are triggered and terminated using the manual triggers.
